# Virtual parking path planning in narrow roads based on fuzzy pure pursuit algorithm

**DOI:** 10.1371/journal.pone.0335911

**Published:** 2025-12-29

**Authors:** Qingyi Men, Yongwei Wang, Guangwei Cheng, Ziyang Zhang, Xuefeng Zhu, Hui Zhou

**Affiliations:** 1 College of Intelligent Vehicle Engineering, Luoyang Institute of Science and Technology, Luoyang, China; 2 School of Vehicle and Traffic Engineering, Henan University of Science and Technology, Luoyang, China; National University of Singapore, SINGAPORE

## Abstract

To address the issues of low adaptability and significant tracking errors in parking scenarios when using fixed look-ahead distance Pure Pursuit (PP) algorithms, this paper proposes an automatic parking path tracking control algorithm based on Fuzzy Pure Pursuit (FPP). Considering the influence of road curvature on look-ahead distance, a fuzzy controller is designed to output speed proportionality coefficient and curvature proportionality coefficient. This enables adaptive adjustment of the look-ahead distance according to vehicle speed and road curvature, thereby enhancing path adaptability and tracking accuracy. Prescan/CarSim/Simulink simulation results demonstrate that in vertical parking scenarios, the FPP-based tracking control algorithm outperforms traditional PP algorithms in tracking performance for desired paths and heading angles. The tracking error is reduced by 4.8%, and the heading angle error is reduced by 7.3%. The test results of the Apollo advanced platform show that, under different initial heading angles, the vehicle is able to successfully track the parking path and completes the parking operation without collisions. The tracking control algorithm based on FPP has excellent environmental adaptability.

## 1. Introduction

With the continuous development of China’s social economy, parking spaces in urban areas are becoming increasingly limited and constrained, making parking operations more difficult [1–2]. Automatic parking technology helps alleviate driver pressure, improve comfort, and ensure safety, thereby attracting widespread attention worldwide [3–5].

Common tracking control methods include Model Predictive Control (MPC) [6–8], Linear Quadratic Regulator (LQR) [9–10], Pure Pursuit (PP), and intelligent control techniques [11–13]. Furthermore, hierarchical architectures [14] and robust model predictive control schemes [15] have been proposed to address coordination and path tracking challenges for connected and automated vehicles, offering advanced solutions for complex scenarios. The PP algorithm selects a preview point on the target path and calculates the necessary steering angle based on the geometric relationship among the vehicle’s current position, the preview point, and the vehicle’s wheelbase. This method is simple and intuitive, computationally efficient, and has low dependence on precise vehicle models, making it highly popular [16–17]. Liu Weidong et al. [18] proposed a road preview model combining path and velocity information, introduced an extended state observer to estimate disturbances from both internal and external environments, and achieved effective compensation, improving the robustness of the PP controller. Qi Zhiquan et al. [19] proposed a memory-parameter-based steering angle correction method to mitigate steering oscillations due to small look-ahead distances. Sun Qinpeng et al. [20] proposed a dual-parameter adjustment method for look-ahead distance based on steering and yaw angles, along with a path expansion suppression strategy using dual-tangent circle correction to reduce large tracking errors in sharp curves. Ahn J et al. [21] applied Dubins curves for parking path planning and designed a heuristic method to dynamically select the preview point based on vehicle-path correlation. This method extrapolates the initial preview point along the normal direction of the path in high-curvature regions to mitigate corner-cutting issues.

These studies show that look-ahead distance directly impacts the tracking performance and is critical for the effectiveness of the PP algorithm. This paper focuses on space-constrained parking scenarios, proposes a fuzzy control-based FPP tracking method that adjusts the look-ahead distance adaptively according to vehicle speed and road curvature, and verifies the algorithm’s performance through simulations and real-vehicle tests on the Apollo platform.

## 2. Kinematic model and path planning

### 2.1. Vehicle kinematic model

**Fig 1:**
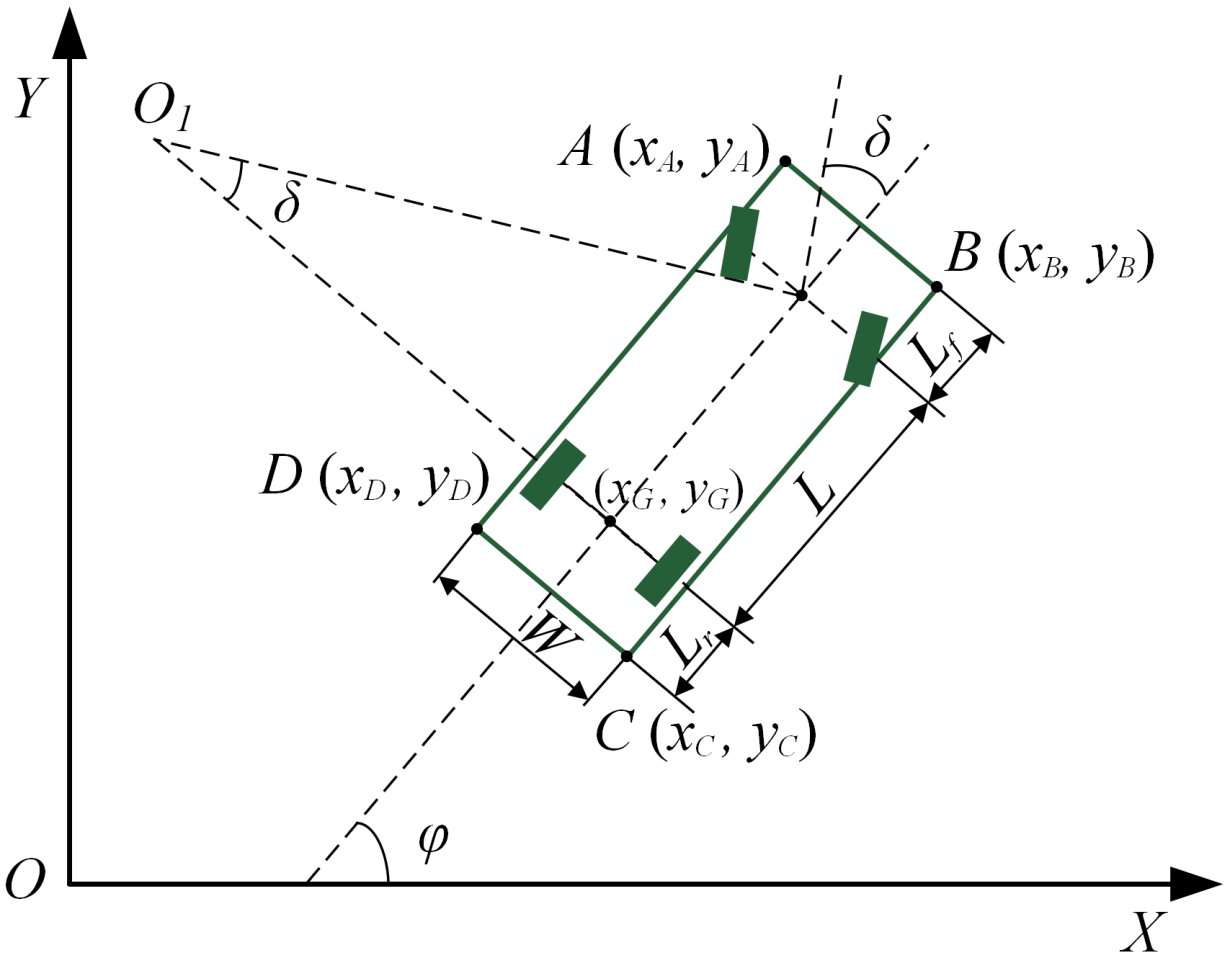
Vehicle kinematic model.

The vehicle outline is described by a rectangle defined by the vehicle’s maximum length and maximum width [22]. Based on the Ackermann steering principle, the kinematic model of the vehicle is established, as shown in Fig 1.

According to the Ackermann steering model, the motion trajectories of the front and rear wheels can be represented by two equivalent wheels located at the centers of the front and rear axles, respectively. The equivalent front wheel steering angle is denoted as *δ*, and the relationship between the front and rear steering angles is simplified as follows:


$$\cot\alpha_{1}+\cot\alpha_{2}=2\cot\delta$$ (1)

In this expression, *α*_2_ represents the inner front wheel steering angle, and *α*_1_ denotes the outer front wheel steering angle. Taking the center of the rear axle as the origin, the kinematic model is established as follows:


$$\left\{ \;\;\;\begin{array}{c} \dot{x}=v_{r}\cos\varphi \\ \dot{y}=v_{r}\sin\varphi \\ \dot{\varphi }=v_{r}\tan\delta /L \end{array}\right.$$ (2)

*φ* is the vehicle heading angle, and *v*_*r*_ is the vehicle speed.

During steering, the perpendicular lines of all wheels intersect on the extension of the rear axle. In this case, based on the center of the rear axle, the turning radius, and vehicle parameters, the positions of the four corners of the vehicle outline can be determined. This allows us to assess whether a collision with an obstacle occurs during the parking process.

### 2.2. Parking scenario model

**Fig 2:**
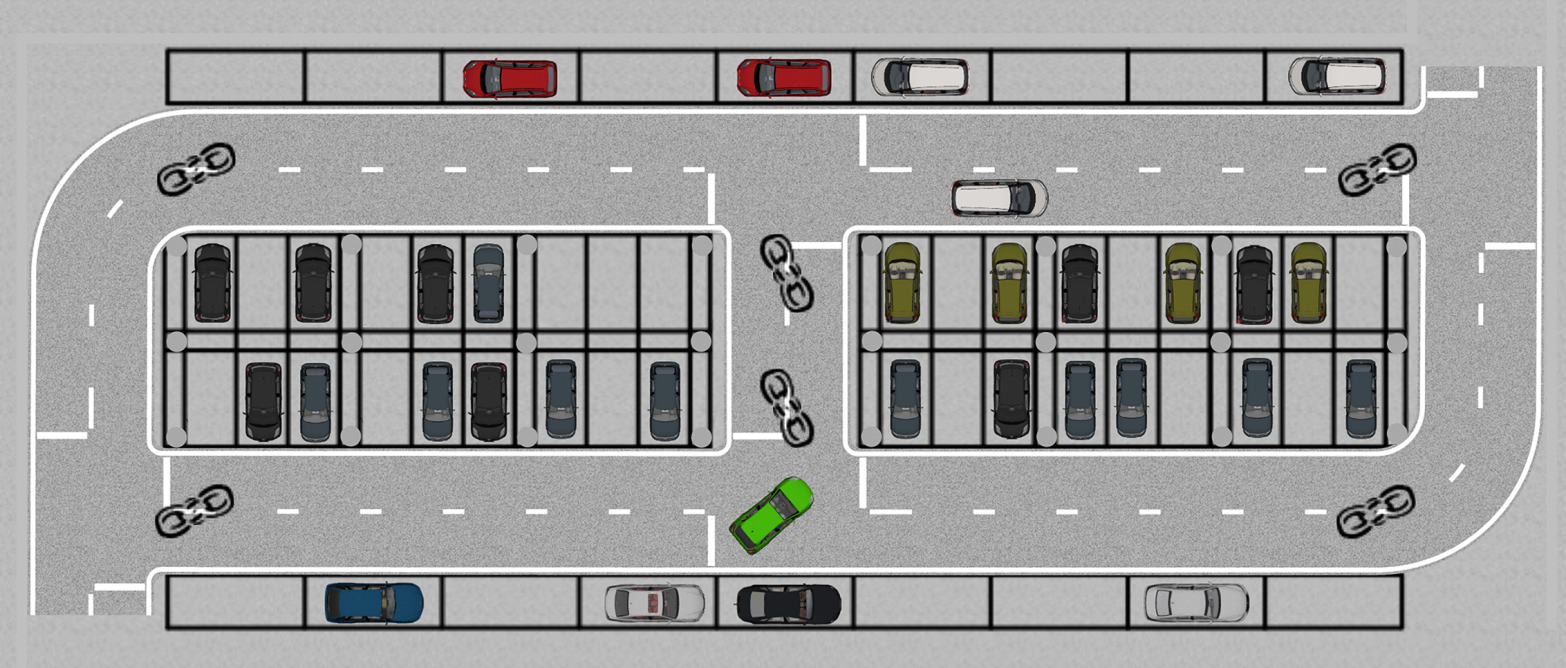
Parking scenario planar model.

Having established the kinematic model, we now define the specific parking scenario in which this model will be applied. Perpendicular parking spaces are commonly found in indoor parking scenarios, which often feature limited space and numerous static obstacles such as pillars. Studying tracking control in such specific scenarios is therefore of particular importance. To this end, an indoor parking lot was constructed in Prescan, including both perpendicular and parallel parking spaces, as shown in Fig 2.

The road width and parking space dimensions in the indoor parking scenario were designed according to the garage building code JGJ 100–2015. Specific vehicle parameters and parking space dimensions are listed in Table 1.

**Table 1:** Vehicle and environment parameters.

Parameter/Unit	Value
Wheelbase *L*/m	2.45
Front overhang *L*_*f*_/m	0.8
Rear overhang *L*_*r*_/m	0.95
Vehicle width *W*/m	1.65
Minimum turning radius *R*_min_/m	4.2
Aisle width *W*_*A*_/m	3
Perpendicular space length *L*_*P*_/m	5.5
Perpendicular space width *W*_*P*_/m	2.5

### 2.3. Perpendicular parking path planning

**Fig 3:**
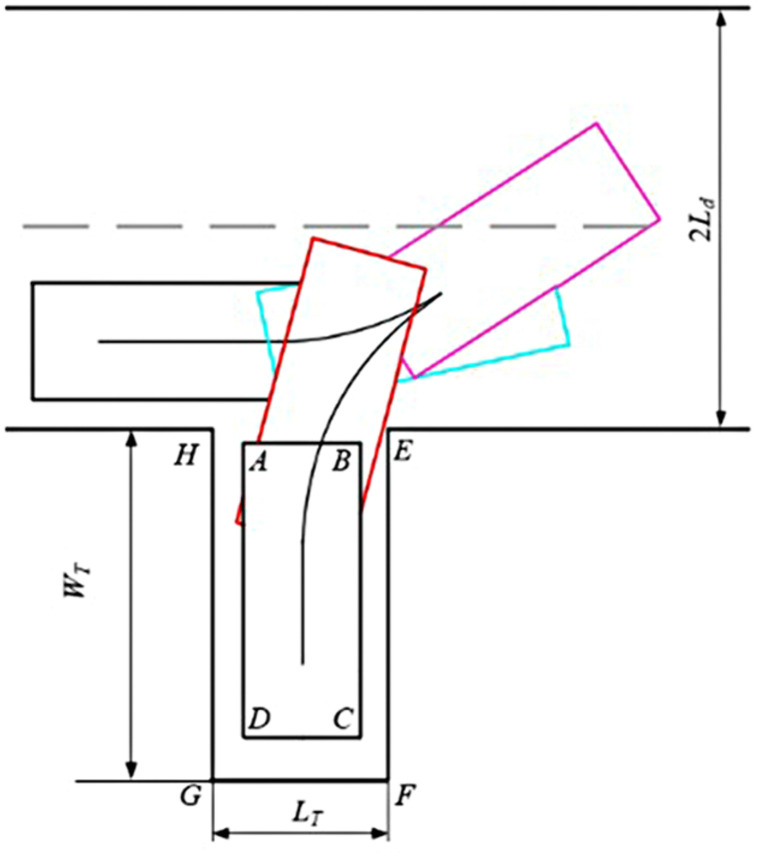
Multi-segment vertical parking path.

During single-step perpendicular parking, the vehicle typically requires a large lateral space, which may lead to parking failure when lateral space is limited. Therefore, a multi-phase forward and backward perpendicular parking path is also designed. The endpoint of the global path serves as the starting point of the preparatory phase. The preparatory phase guides the vehicle to the starting point of the parking phase with a large orientation angle. The parking phase then guides the vehicle into the parking space, as shown in Fig 3. Define that the Preparatory phase means the phase that guides the vehicle to the starting point of the parking phase with a large orientation angle. The Parking-in phase:the phase that guides the vehicle from the starting point into the parking space.

When using circular arcs to plan parking paths, curvature discontinuities at the arc junctions can lead to in-place steering, increasing tire wear and steering motor load. As a type of transition curve, the clothoid is widely used in road and railway design. When a vehicle moves with constant longitudinal and steering velocities, the resulting path is a clothoid curve [23]. By introducing a clothoid between circular arcs with opposite curvatures, overall curvature continuity can be maintained. Let the curvature rate of the clothoid be *k*, with the initial point fixed at the origin and the initial heading angle set to zero. Then, the coordinate (*x*, *y*) and heading angle *ρ* of the point on the clothoid where the curvature *α*_0_ is given by:


$$x=\sum_{i=0}^{N}{\left(-1\right)}^{i}\frac{\rho^{4i+1}}{2^{2i}k^{2i+1}\left(4i+1\right)\left(2i\right)!}$$ (3)

$$y=\sum_{i=0}^{N}{\left(-1\right)}^{i}\frac{\rho^{4i+3}}{2^{2i+1}k^{2i+2}\left(4i+3\right)\left(2i+1\right)!}$$ (4)

$$\alpha_{0}=\frac{\rho^{2}}{2k}$$ (5)

In this expression, *N* is the power series expansion coefficient. In this work, we set *N* = 15.

## 3. Parking path tracking control

### 3.1. Principle of the Pure Pursuit (PP) algorithm

**Fig 4:**
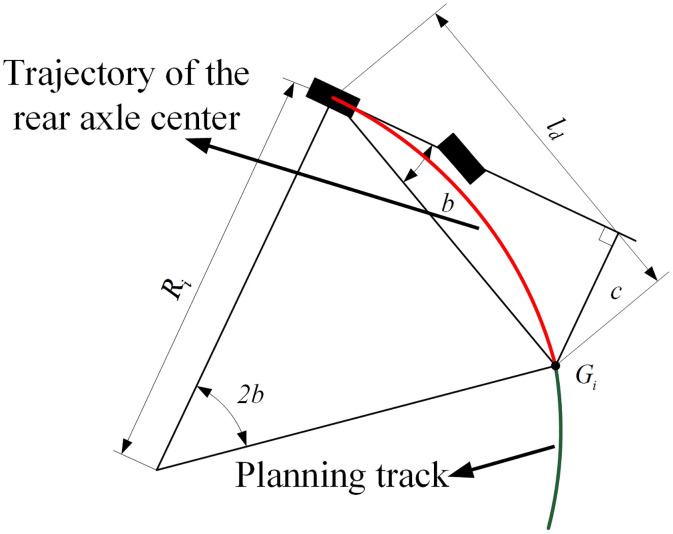
Geometric description of PP algorithm.

The PP algorithm provides good tracking performance under low-speed conditions. Its geometric representation is shown in Fig 4. Here, *G*_*i*_ is the look-ahead point, *l*_*d*_ is the look-ahead distance, *b* is the angle between the vehicle’s longitudinal axis and the line *G*_*i*_ connecting the rear axle center to the look-ahead point, and *R*_*i*_ is the radius of the desired path arc. Taking the center of the rear axle as the tangent point and the vehicle’s longitudinal axis as the tangent line, the front wheel steering angle is controlled to drive the vehicle along a curve that passes through the look-ahead point, thereby achieving path tracking control.

Based on the geometric relationship, the curvature of the desired path can be obtained as follows:


$$\frac{l_{d}}{\sin\left(2b\right)}=\frac{R_{i}}{\sin\left(\frac{\pi }{\text{2}}-b\right)}$$ (6)

Based on the kinematic vehicle model established in the previous section, the front wheel steering angle control law of the PP algorithm can be expressed as:


$$\delta_{p}=\tan^{-1}(\frac{2L\sin b}{l_{d}})$$ (7)

The calculation method for the look-ahead distance is:


$$l_{d}=l+\tau v$$ (8)

**Fig 5:**
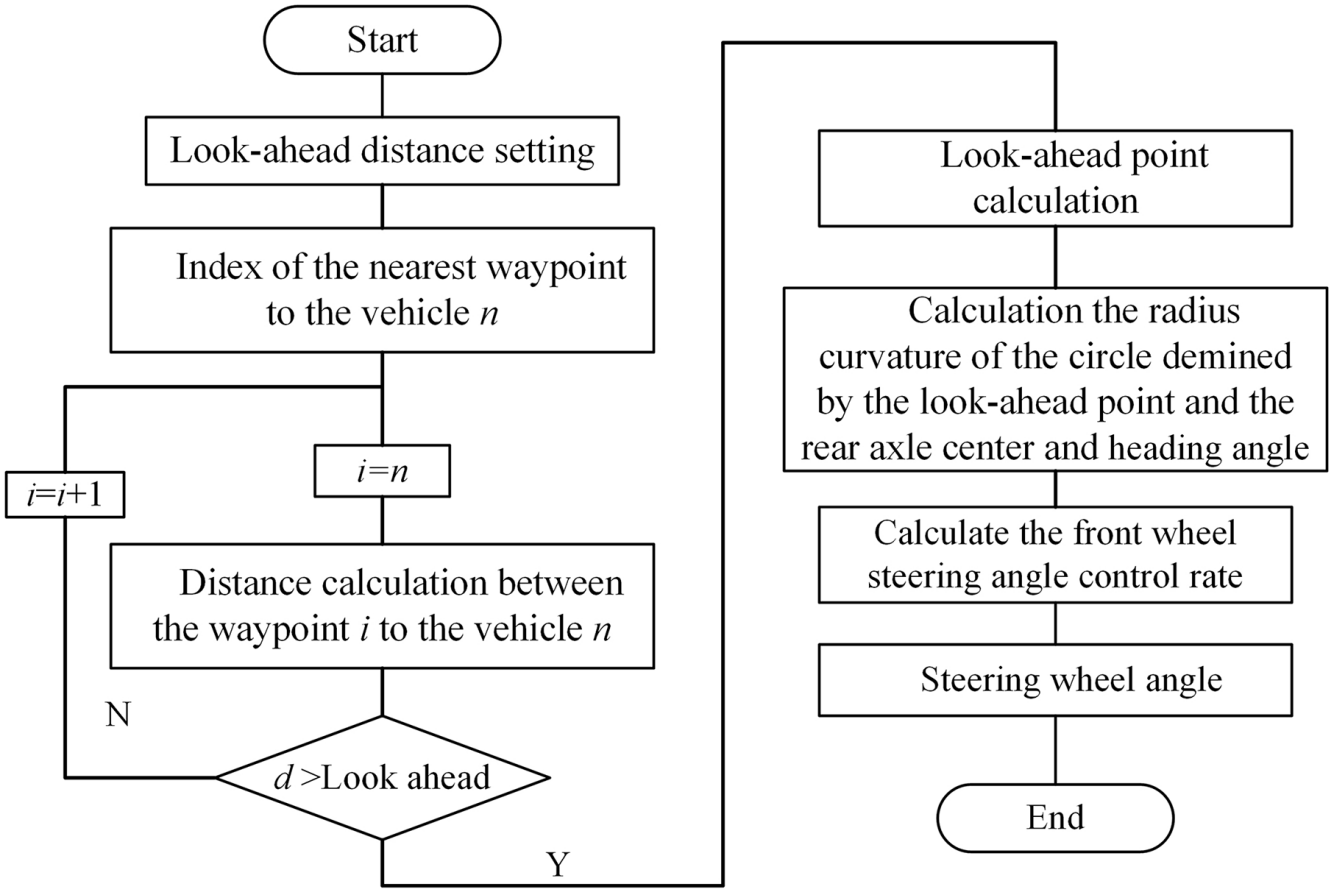
PP algorithm flowchart.

In this expression, *l* is the minimum look-ahead distance, determined by the parking driving condition; *τ* is the velocity proportional coefficient; and *v* is the longitudinal velocity of the vehicle. Given a specified look-ahead distance, the corresponding equivalent front wheel steering angle can be calculated for each *b*, and the corresponding steering wheel angle is obtained using the steering ratio. This angle is then used as the control input to achieve path tracking. The algorithm flowchart is shown in Fig 5.

### 3.2. Adaptive adjustment of look-ahead distance

**Fig 6:**
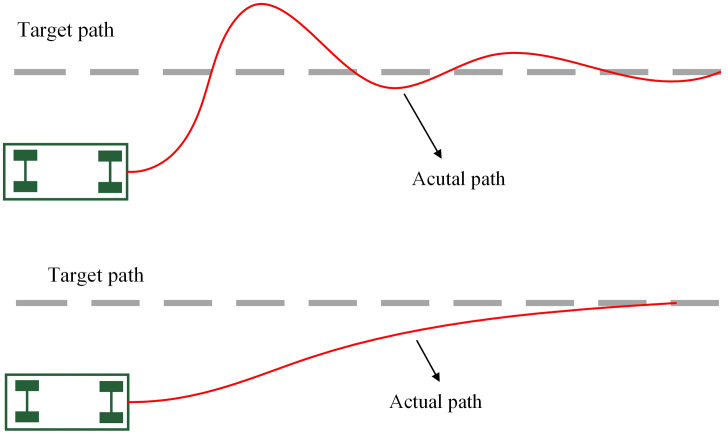
Look-ahead distance impact on path.

The tracking accuracy of the PP algorithm is directly affected by the look-ahead distance. If the selected look-ahead distance is too short, frequent steering can cause control oscillations and reduced comfort. If the look-ahead distance is too long, oscillations are reduced, but the control system may ignore parts of the reference path, resulting in shortcut behaviors such as premature turning and reduced tracking accuracy, as shown in Fig 6.

Traditional methods for determining the look-ahead distance consider only vehicle speed, which can lead to large tracking errors when driving on high-curvature paths. Therefore, the influence of road curvature is incorporated into the design of the look-ahead distance. The improved calculation method for the look-ahead distance is:


$$l_{d}^{\prime }=l+\tau v-\tau_{c}g^{\prime }$$ (9)

In this expression, *τ*_*c*_ is the curvature proportional coefficient, and g’ is the curvature at the look-ahead point.

When the road curvature is high, a smaller look-ahead distance is selected; when the curvature is low, a larger look-ahead distance is chosen. Dynamically adjusting the look-ahead distance according to different parking scenarios improves overall path tracking efficiency and safety.

### 3.3. Fuzzy controller design

The adjustment of the proportional coefficients *τ* and *τ*_*c*_ is key to achieving adaptive look-ahead distance control. Fuzzy control is a method that mimics human reasoning and performs judgment using fuzzy mathematics. Based on human experience and knowledge, a fuzzy rule base is established. The vehicle state information is used as the input to the fuzzy controller, and through the processes of fuzzification, fuzzy inference, and defuzzification, a precise target output is obtained [24].

The dynamic look-ahead distance aims to address the limited adaptability of fixed look-ahead strategies in complex indoor parking scenarios. The design concept is as follows:

1. Dynamically adjust the look-ahead baseline according to vehicle speed—shortening the look-ahead distance at low speeds to improve response time and extending it at higher speeds to suppress oscillations.
2. Modify the look-ahead distance based on curvature variation along curved paths—reducing or increasing it dynamically to allow the vehicle to adjust its heading in advance before entering a curve.

Based on this, a fuzzy controller is designed. The output logic for *τ* follows a low-speed large-deviation to high-speed small-deviation principle. Specifically, vehicle speed *v* and lateral deviation *e*_*h*_ are used as inputs: when *v* is small and *e*_*h*_ is large, the value of *τ* is increased; when *v* is large and *e*_*h*_ is small, the value of *τ* is decreased. The output logic for *τ*_*c*_ follows a sharp-turn large-heading-deviation to straight-path small-heading-deviation principle. Road curvature *ρ* and heading deviation *e*_*θ*_ are used as inputs: when both *ρ* and *e*_*θ*_ are large, the value of *τ*_*c*_ is increased; when bo*th ρ* are *e*_*θ*_ small, the value of *τ*_*c*_ is decreased. The control variables and their universes of discourse are listed in Table 2.The input variables were selected because they directly represent the primary state errors in path tracking. Lateral deviation reflects the positional inaccuracy, while heading deviation reflects the angular inaccuracy. Although the curvature rate is also an important factor, it is indirectly accounted for by the road curvature and its effect on the heading deviation. This choice strikes a balance between controller complexity and performance, focusing on the most salient features for parking maneuvers.

**Table 2:** Control variables and universes of discourse.

Control Variables	Universes of Discourse
*e*_*h*_	[-0.5m, 0.5m]
*v*	[-0.833m/s, 0.833m/s]
*e*_*θ*_	[-0.4rad, 0.4rad]
*ρ*	[-0.5m^-1^, 0.5m^-1^]
*τ*	[0.2, 1.5]
*τ*_*c*_	[0.1, 0.8]

**Fig 7:**
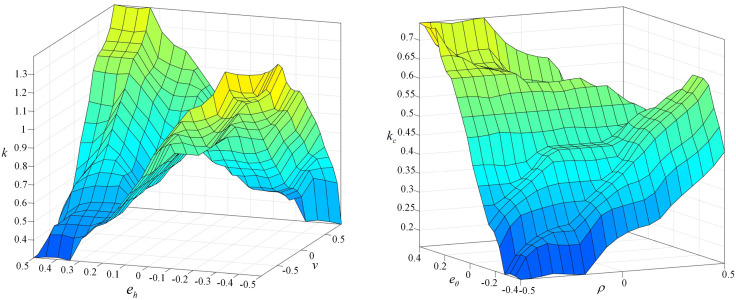
3D surface graphs of the fuzzy rules.

The fuzzy linguistic values for lateral deviation *e*_*h*_ and heading deviation *e*_*θ*_ are [FD, FZ, FX, Z, ZX, ZZ, ZD], representing large negative, medium negative, small negative, zero, small positive, medium positive, and large positive, respectively. The fuzzy linguistic values for heading deviation *e*_*θ*_ have the same meanings as those for lateral deviation *e*_*h*_. The fuzzy linguistic values for vehicle speed *v* are [NF, NL, ZO, PL, PF], representing fast negative, slow negative, zero, slow positive, and fast positive, respectively. The fuzzy linguistic values for road curvature *ρ* are [NB, NS, ST, PS, PB], representing sharp left turn, slight left turn, straight road, sharp right turn, and slight right turn, respectively. The fuzzy linguistic values for the speed and curvature proportional coefficients *τ* and *τ*_*c*_ are [LP, SP, H, SN, LN], representing very small, small, medium, large, and very large. The fuzzy rules for *τ* and *τ*_*c*_ are shown in Tables 3 and 4, and the fuzzy rule surfaces are illustrated in Fig 7.

**Table 3:** Fuzzy rules for *τ.*

*τ*		Lateral Deviation *e*_*h*_						
		FD	FZ	FX	Z	ZX	ZZ	ZD
speed *v*	NF	LP	LP	SP	H	SN	LN	LN
	NL	LP	SP	H	H	SN	SN	LN
	ZO	SP	H	H	H	SN	H	SN
	PL	H	SN	SN	H	H	SP	SP
	PF	LN	LN	SN	H	SP	LP	LP

**Table 4:** Fuzzy rules for *τ*_*c.*_

*τ*_*c*_		Course Deviation *e*_*θ*_						
		FD	FZ	FX	Z	ZX	ZZ	ZD
Curvature *ρ*	NB	LN	LN	SN	H	SP	LP	LP
	NS	LN	SN	H	H	SP	SP	LP
	ST	SN	H	H	H	SP	H	SP
	PS	H	SP	SP	H	H	SN	SN
	PB	SP	LP	LP	H	LN	LN	LN

Based on the above rules, fuzzy inference is performed using the Mamdani method, and defuzzification is carried out using the centroid method to obtain the final control outputs *τ* and *τ*_*c*_.

### 3.4. FPP lateral controller architecture

**Fig 8:**
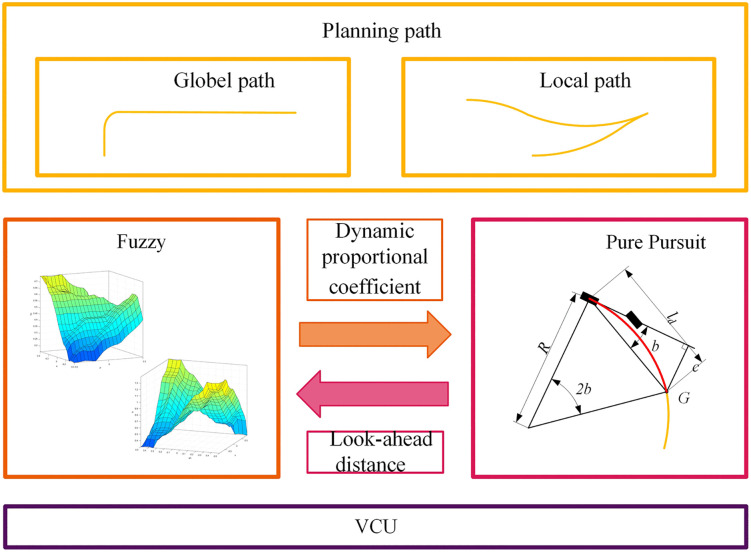
FPP lateral controller architecture.

Based on the PP algorithm improved by fuzzy control, the FPP lateral control architecture is designed as shown in Fig 8. This architecture outputs a dynamic speed proportional coefficient *τ* and curvature proportional coefficient *τ*_*c*_ through the fuzzy control algorithm. The look-ahead distance is adaptively adjusted according to the vehicle speed *v* and road curvature *ρ*. Finally, the pure pursuit module calculates the adaptive look-ahead distance using Equation (9).

The FPP architecture effectively integrates traditional and advanced control strategies to achieve full-condition adaptive look-ahead distance adjustment, providing a robust and stable solution for autonomous parking path tracking.

## 4. Co-simulation validation

**Fig 9:**
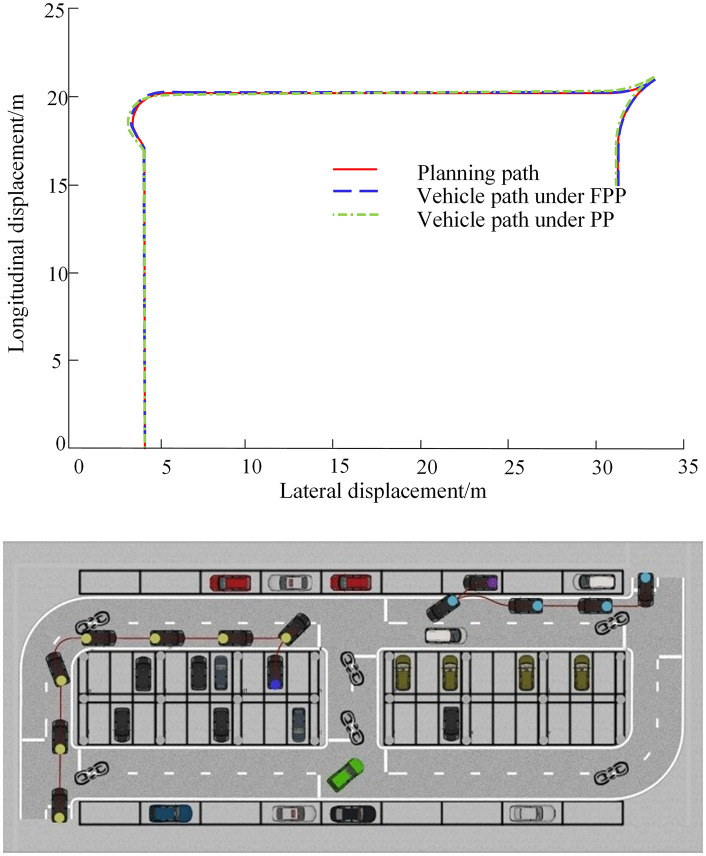
Vertical parking tracking in co-platform.

In the constructed Prescan scenario, a comparative simulation was conducted between the proposed FPP path tracking controller and the fixed look-ahead distance PP controller. The path tracking performance under perpendicular parking conditions in the co-simulation platform is shown in Fig 9.

**Fig 10:**
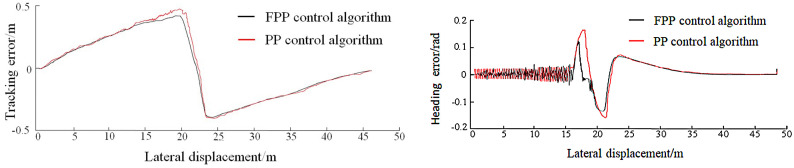
Global path tracking error and heading error.

**Fig 11:**
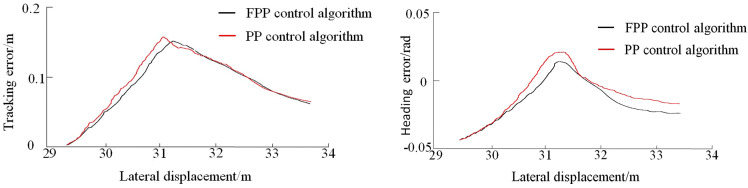
Preparatory phase path tracking error and heading error.

**Fig 12:**
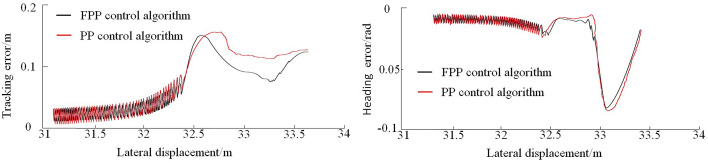
Parking-in phase path tracking error and heading error.

As shown in Fig 9, the FPP lateral controller accurately guides the vehicle to track the desired path and reach the target parking space with the expected pose, completing the parking operation. The tracking error and heading angle error for each path segment during the parking process are compared in Figs 10–12.

As shown in Figs 10–12, the tracking error under the global path is primarily concentrated at the steering sections. During the parking-in phase, slight vehicle body oscillations are observed. Due to the accumulation of errors, the tracking error on the local path is slightly higher than that on the global path. At the end of the parking task, a small deviation in the vehicle’s heading angle remains. The maximum tracking error and heading angle error for each path segment under both control methods are summarized in Table 5.

**Table 5:** The maximum tracking error and heading angle error.

vehicle tracks	The Maximum Tracking Error/m	The Maximum Heading Angle Error/rad
FPP Global path	0.392	0.133
FPP Preparation section	0.146	0.027
FPP Berthing section	0.152	0.081
FPP Endpoint	0.019	0.007
PP Global path	0.489	0.181
PP Preparation section	0.152	0.044
PP Berthing section	0.156	0.084
PP Endpoint	0.022	0.011

Analysis of the statistical results shows that the FPP algorithm achieves lower maximum tracking and heading angle errors across all path segments compared to the traditional PP algorithm. Overall, the tracking error is reduced by 4.8%, and the heading angle error is reduced by 7.3%. This indicates that the FPP algorithm effectively avoids increased tracking error during steering, offering high tracking accuracy and good applicability.

## 5. Apollo advanced platform test

**Fig 13:**
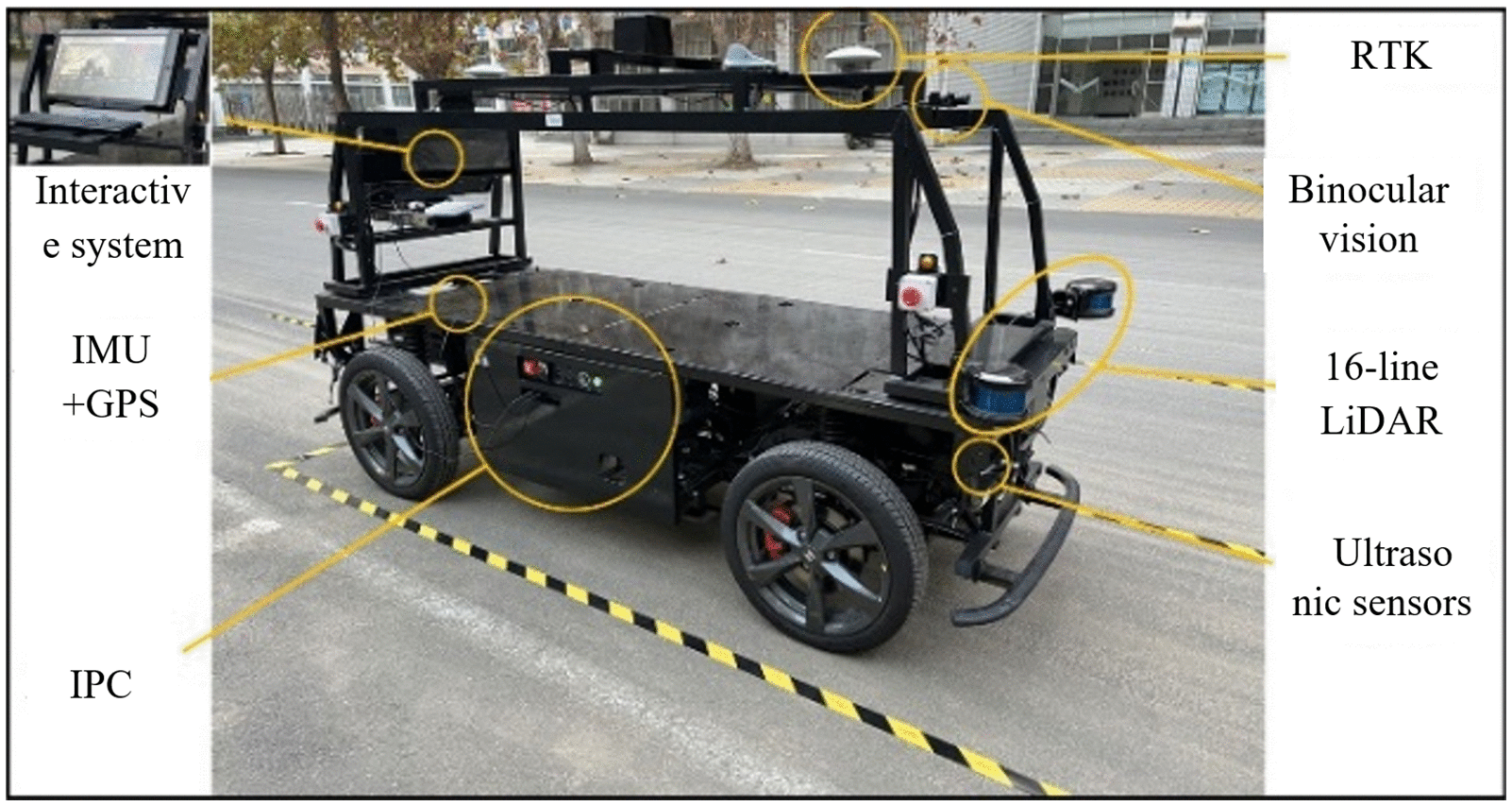
Apollo advanced D-Kit.

To further investigate the applicability of the proposed FPP algorithm, an experiment was designed using the Apollo Advanced platform, as shown in Fig 13. This platform is equipped with LiDAR, ultrasonic sensors, and a GPS + IMU integrated navigation system, enabling basic parking experiments [25]. The tracking module is constructed using a lateral pure pursuit algorithm and a longitudinal dual-PID controller. Steering and acceleration/deceleration commands are executed by a drive-by-wire chassis to guide the vehicle into the parking space. The record function is used to log experimental data and complete the parking operation. The basic parameters of the platform are listed in Table 6.

**Table 6:** Apollo advanced parameters.

Parameter/Unit	Value
Wheelbase/m	1.730
Curb weight/kg	420
CG to Front Axle Distance/m	0.888
CG to Rear Axle Distance/m	0.892

**Fig 14:**
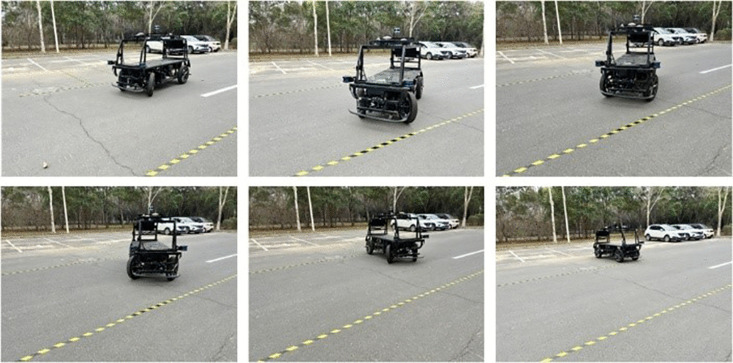
Automated parking process for vertical parking.

**Fig 15:**
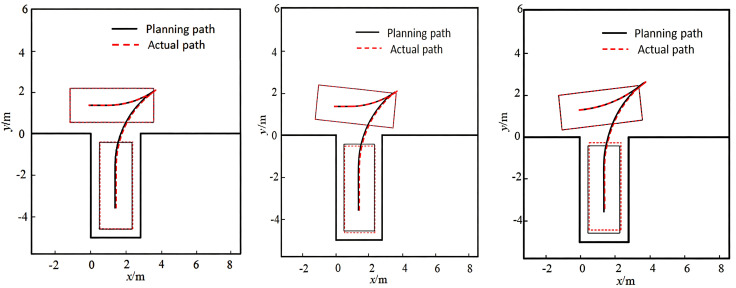
Vertical parking paths under different initial angles.

To simulate a typical parking environment, the vehicle speed was limited to no more than 1 m/s. Based on the scale ratio between the Apollo vehicle and a real vehicle, and the dimensions of a standard parking space, the target space was set to 4.53 m in length and 1.41 m in width, with a double-lane parking aisle width of 3.96 m. The vehicle reaching the target parking point serves as the termination trigger. The experimental procedure is illustrated in Fig 14. Tests were conducted with the initial vehicle pose set to 0°, 5°, and −5°, respectively. The results are shown in Fig 15.

As shown in Fig 15, the vehicle successfully tracks the parking path and completes the parking operation without collisions under different initial heading angles. Due to the influence of tracking error and initial angle, there is a certain longitudinal deviation between the final parking position and the planned target point. In the case of an initial angle of 5°, the vehicle ends closer to the lower boundary of the parking space, while in the case of −5°, the vehicle ends closer to the right side. These results demonstrate good environmental adaptability.

## 6. Conclusion

(1) To address the low adaptability and large tracking error associated with fixed look-ahead distance pure pursuit control in parking scenarios, an FPP (Fuzzy-based Pure Pursuit) control algorithm is proposed. This algorithm incorporates road curvature into the look-ahead distance calculation and employs a fuzzy controller to output the speed and curvature proportional coefficients, enabling adaptive adjustment of the look-ahead distance based on vehicle speed and road curvature. Simulation and experimental results show that the proposed FPP algorithm accurately tracks the planned path under perpendicular parking conditions, with a maximum tracking error of 0.392 m and a maximum heading angle error of 0.133 rad, demonstrating significant improvements in adaptability and tracking accuracy.
(2) Real-vehicle experiments were conducted to further verify the effectiveness of the proposed FPP algorithm. Three different initial poses were tested, and in all cases the vehicle successfully tracked the parking path without collisions, completing the parking operation successfully and demonstrating good applicability.

In real scenarios, vehicles also encounter parallel and angled parking spaces. Future work will focus on developing tracking control methods for these two parking types.

## Supporting information

S1 Appendix Global path tracking process data for each path segment under the two control methods. (DOCX)
