# Supplementary material for: Virtual parking path planning in narrow roads based on fuzzy pure pursuit algorithm
Source: PLoS One. 2025 Dec 29;20(12):e0335911. doi: 10.1371/journal.pone.0335911 (PMC12747341; doi:10.1371/journal.pone.0335911)
Supplement: S1 Appendix — (DOCX) [file pone.0335911.s001.docx]

**Global path tracking process data for each path segment under the two control methods**

**Data from the Testing Process is as follows:**

**Table 1. Global Path Tracking Process Error Analysis (FPP Global path)**

| **Path Point Description** | **Planned Heading Angle (rad)** | Actual Heading Angle (rad) | **Heading Angle Error (rad)** | Lateral Tracking Error (m) |
| --- | --- | --- | --- | --- |
| **Phase Start** | 0.000 | 0.000 | 0.000 | 0.000 |
| **First Curve Entrance** | 0.100 | 0.120 | 0.020 | 0.045 |
| **First Curve Midpoint** | **0.350** | **0.380** | **0.030** | **0.120** |
| **First Curve Exit** | 0.150 | 0.170 | 0.020 | 0.095 |
| **Second Curve Entrance** | -0.120 | -0.100 | 0.020 | 0.110 |
| **Second Curve Midpoint** | **-0.400** | **-0.450** | **0.050** | **0.280** |
| **Path End Point** | 0.000 | 0.010 | 0.010 | 0.019 |
| **Maximum (Max)** | — | — | **0.133** | **0.392** |

**Table 2. Preparation Phase Path Tracking Process Error Analysis (FPP Preparation section)**

| **Path Point Description** | **Planned Heading Angle (rad)** | Actual Heading Angle (rad) | **Heading Angle Error (rad)** | Lateral Tracking Error (m) |
| --- | --- | --- | --- | --- |
| **Phase Start** | 0.000 | 0.000 | 0.000 | 0.000 |
| **Midpoint of Straight** | 0.000 | 0.000 | 0.000 | 0.015 |
| **Steering Start** | 0.050 | 0.060 | 0.010 | 0.035 |
| **Steering Midpoint** | **0.200** | **0.220** | **0.020** | **0.095** |
| **Phase End Point** | 0.100 | 0.110 | 0.010 | 0.146 |
| **Maximum (Max)** | — | — | **0.027** | **0.146** |

**Table 3. Berthing Phase Path Tracking Process Error Analysis (FPP Berthing section)**

| **Path Point Description** | **Planned Heading Angle (rad)** | Actual Heading Angle (rad) | **Heading Angle Error (rad)** | Lateral Tracking Error (m) |
| --- | --- | --- | --- | --- |
| **Phase Start** | 0.100 | 0.110 | 0.010 | 0.146 |
| **First Adjustment Point** | 0.250 | 0.280 | 0.030 | 0.130 |
| **Steering Reversal Point** | **-0.150** | **-0.080** | **0.070** | **0.140** |
| **Second Adjustment Point** | -0.300 | -0.250 | 0.050 | 0.125 |
| **Body Aligning Point** | **-0.100** | **-0.030** | **0.070** | **0.152** |
| **Phase End Point** | 0.000 | 0.007 | 0.007 | 0.019 |
| **Maximum (Max)** | — | — | **0.081** | **0.152** |

**Table 4. Global Path Tracking Process Error Analysis (PP Global path)**

| **Path Point Description** | **Planned Heading Angle (rad)** | Actual Heading Angle (rad) | **Heading Angle Error (rad)** | Lateral Tracking Error (m) |
| --- | --- | --- | --- | --- |
| **Phase Start** | **0.000** | **0.000** | **0.000** | **0.000** |
| **First Curve Entrance** | **0.100** | **0.060** | **0.040** | **0.100** |
| **First Curve Lag Point** | **0.350** | **0.250** | **0.100** | **0.350** |
| **First Curve Exit** | **0.150** | **0.180** | **0.030** | **0.250** |
| **Second Curve Entrance** | **-0.120** | **-0.050** | **0.070** | **0.300** |
| **Second Curve Lag Point** | **-0.400** | **-0.250** | **0.150** | **0.489** |
| **Path End Point** | **0.000** | **-0.050** | **0.050** | **0.200** |
| **Maximum (Max)** | **—** | **—** | **0.181** | **0.489** |

**Table 5. Preparation Phase Path Tracking Process Error Analysis (PP Preparation section)**

| **Path Point Description** | **Planned Heading Angle (rad)** | Actual Heading Angle (rad) | **Heading Angle Error (rad)** | Lateral Tracking Error (m) |
| --- | --- | --- | --- | --- |
| **Phase Start** | **0.000** | **0.000** | **0.000** | **0.000** |
| **Midpoint of Straight** | **0.000** | **0.005** | **0.005** | **0.030** |
| **Steering Start** | **0.050** | **0.020** | **0.030** | **0.080** |
| **Steering Midpoint** | **0.200** | **0.160** | **0.040** | **0.140** |
| **Phase End Point** | **0.100** | **0.130** | **0.030** | **0.152** |
| **Maximum (Max)** | **—** | **—** | **0.044** | **0.152** |

**Table 6. Berthing Phase Path Tracking Process Error Analysis (PP Berthing section)**

| **Path Point Description** | **Planned Heading Angle (rad)** | Actual Heading Angle (rad) | **Heading Angle Error (rad)** | Lateral Tracking Error (m) |
| --- | --- | --- | --- | --- |
| **Phase Start** | **0.100** | **0.130** | **0.030** | **0.152** |
| **First Adjustment Lag Point** | **0.250** | **0.180** | **0.070** | **0.148** |
| **Steering Reversal Point** | **-0.150** | **-0.050** | **0.100** | **0.155** |
| **Second Adjustment Lag Point** | **-0.300** | **-0.180** | **0.120** | **0.145** |
| **Body Aligning Point** | **-0.100** | **0.020** | **0.120** | **0.156** |
| **Phase End Point** | **0.000** | **0.011** | **0.011** | **0.022** |
| **Maximum (Max)** | **—** | **—** | **0.084** | **0.156** |

As can be seen from Table 1, the FPP algorithm, by adapting the look-ahead distance, can closely track the path. Errors are mainly concentrated at the midpoints of curves with the highest curvature, and the overall error is controllable. From Table 2, it can be observed that during the preparation phase, the FPP algorithm responds quickly to steering commands, with both heading and lateral errors increasing steadily. Table 3 shows that during the berthing phase, where the direction changes repeatedly, the FPP algorithm can adjust dynamically. However, significant heading angle error still occurs momentarily at steering reversal instants but is quickly corrected.

From Table 4, it can be seen that the PP algorithm exhibits understeer at curve entrances due to the look-ahead point being too far (Actual Heading Angle < Planned Heading Angle). A "catch-up" effect appears in the mid-to-late part of the curve, but it is too late, causing the maximum error point to "lag" behind the point of maximum curvature. Table 5 shows that the PP algorithm responds sluggishly at the start of steering (error already reaches 0.030 rad), and overshoots at the end, resulting in an excessive heading angle. This reflects the imprecision of fixed look-ahead distance control on dynamically changing paths. Table 6 indicates that the hysteresis of the PP algorithm is amplified during the berthing phase requiring precise positioning. It is constantly "one step behind," failing to achieve the required large heading angle when planned and failing to realign properly when needed, resulting in consistently high heading angle errors throughout the phase. Its control performance is significantly inferior to FPP.

The above data demonstrate that the maximum errors typically occur during dynamic stages such as curve midpoints or steering reversal points. However, the FPP controller can effectively keep the errors within the expected range.
